# Knowledge and practice of immediate newborn care among health care providers in eastern zone public health facilities, Tigray, Ethiopia, 2016

**DOI:** 10.1186/s12887-017-0915-8

**Published:** 2017-07-11

**Authors:** Abadi Kidanemariam Berhe, Fitiwi Tinsae, Gebremedhin Gebreegziabher

**Affiliations:** 1 Adigrat University, College of Medicine and Health Sciences , Adigrat, Ethiopia; 2 Department of Nursing, College of Medicine and Health Sciences, Adigrat University, Adigrat, Ethiopia; 3 Department of Midwifery, College of Medicine and Health Sciences, Adigrat University, Adigrat, Ethiopia

**Keywords:** Knowledge, Practice, Immediate newborn care, Tigray, Ethiopia

## Abstract

**Background:**

According to WHO (2013) report the number of under five-year mortality in Ethiopia was 195,504, out of this 84,437 was from neonatal death and this mortality is related to immediate obstetric and newborn care of babies provided by health care providers; But little was known about the level of knowledge and practice related to immediate newborn care and their associated factors among health care providers generally in Tigray region and specifically in the Eastern Zone so the aim of this study was to assess knowledge and practice of immediate newborn care and associated factors among health care providers in the Eastern zone public health facilities, Tigray, Ethiopia.

**Methods:**

A cross-sectional study was conducted from December 2015 to February 2016. A total of 16 health care facilities were selected for study using simple random sampling techniques and all health care providers in the selected health care facilities who participated in immediate newborn care were involved in the study. Data were entered, cleaned and analyzed using SPSS version 20.0. Ethical clearance was obtained from Adigrat University institutional ethical review board and Tigray regional health bureau. Consent was obtained from participants to conduct the study.

**Result:**

In this study 215 participants were contacted, with a response rate of 99.1%. Generally, from the health care providers who participated in this study, 74.65% had adequate knowledge on newborn care and overall 72.77% of the participants were having good newborn care practice. Among the health care providers participated in the study, 151 (70.9%) were getting access to newborn care national guideline and only 99 (46%) of the health care providers get training in newborn care within the past two years before the study. Availability of national guideline, having adequate materials, the period of taking training and type of health facility were significant predictors for the health care providers newborn care practice.

**Conclusion:**

Even though some improvement observed in the knowledge and practice of health care providers on newborn care, but still this study identified knowledge and practice gap. Regional health bureau and district health offices should provide refreshment training on immediate newborn care regularly, equipping all health facilities with necessary materials and national guideline of newborn care and there should be sharing experience between hospital and health center staffs working on newborn care through mentoring.

## Background

According to WHO 2015 report globally every year, 2.7 million neonates die during the neonatal period which constitutes 45% of under-5 mortality and approximately 58% of infant mortality and 75% of the deaths occur during the first week in the neonatal period [1, 2]. Most neonatal deaths were in low and middle-income countries, which account for a growing proportion of all under-five mortality [3]**.** If the trend continues like this the share of neonatal deaths to under-five death is projected to increase from 45% in 2015 to 52% in 2030 [4]. Similarly, according to WHO (2013) report, the number of under five-year mortality in Ethiopia was 195,504, out of this 84,437 was from neonatal death [5]. According to the 2011 Ethiopia Demographic and Health Surveys (EDHS), the neonatal mortality rate was 37 per 1000 live births and the perinatal mortality rate was 46 per 1000 this is high compared to the African region average neonatal mortality rate which was 35.9 per 1000 live births [6].

A study conducted in Ethiopia on trend and determinants of neonatal mortality identified sepsis, asphyxia, birth injury, tetanus, preterm birth, congenital malformations as a cause of early neonatal mortality [7]. Immediate newborn care interventions are part of essential newborn care used to protect against newborn morbidity and mortality by using clean cord care, thermal care including drying and wrapping of the newborn immediately after delivery and delaying the newborn’s first bath for at least 24 h or several days to reduce hypothermia risk), and initiation of breastfeeding within the first one hour of birth, management of immediate asphyxia, management of early sepsis [8]. Immediate newborn care aims at addressing poor care practices immediately following delivery [9],

Approximately 50 million were dying each year in the late 1980s, but two-thirds of this has been saved through the application of immediate newborn care [10].

Wide acknowledgments were showed that the MDG 4 target for child survival cannot be achieved without a particular focus on newborn health, especially during the first seven days of life [11]. Newborn complications resulting from hypothermia, infection, birth asphyxia and low birth weight that occurs within the first seven days following birth contribute to the highest burden of morbidity and mortality [12, 13].

A study conducted in Uganda among health workers practice with immediate newborn care showed that appropriate implementation is dependent upon levels of knowledge [14]. A similar study conducted in India showed a high level of knowledge among community health workers was considered as very important for improving coverage and adherence to recommended newborn care practices [15]. Other studies conducted in eastern Uganda also showed that neonatal mortality autopsies indicated the low levels of knowledge among health workers regarding prenatal and newborn care as a major determinant of death [16]. A similar study in Uganda showed that 52% of deliveries take place at the health facility, and neonatal mortality remains relatively high at 29 per 1000 live births this might be related to the immediate newborn care and obstetric care [17]. Critically ill newborn babies often present for care at the formal health facilities where general nurses and midwives are routinely deployed [18]. Studies showed that in the neonatal period more than 70% of the current deaths could be prevented through evidence-based procedures (e.g. exclusive breastfeeding and hypothermia management) [19].

Generally, Ethiopia like other countries in the sub-Saharan region has a high perinatal mortality and specifically the study area Tigray regional state is among the top three regional states with a high burden of perinatal mortality and second highest stillbirth from all regional states of Ethiopia according to EDHS 2011.Perinatal deaths have multi-factorial etiology, therefore, to address the high perinatal mortality there is a need for careful analysis of contributing factors in order to design appropriate interventions. Identifying the level of knowledge and practice was important for deciding the implementation strategy and for effective and sustainable implementation of knowledge into practice but only a few studies has evaluated strategies for knowledge translation in low-income countries [1]. Similarly, little is known about the level of knowledge and practice related to immediate newborn care and associated factors among health care worker providing service in intrapartum and postnatal care generally in Tigray region and specifically in Eastern Zone.

Moreover, this study will be used as a baseline data for district health office, human resource of regional health bureau to provide capacity building to health care providers based on the identified gap and used as a baseline for researchers of the further study. So, the aim of this study was to assess knowledge and practice of immediate newborn care and associated factors among health care providers in Eastern zone public health facilities, Tigray, North Ethiopia 2016.

## Methods

A cross-sectional study design was conducted from December 2015 to February 2016 in Eastern zone of Tigray Region**;** to assess knowledge and practice of immediate newborn care and associated factors among health care providers.

According to the 2014 report of Tigray region health bureau, health service coverage in the eastern zone is approximately 80%, with two general hospitals, 4 primary hospital, 26 health centers and 116 health posts. Midwives, Nurses, and public health officers are responsible for providing basic delivery and postnatal service both in hospitals and health centers in the study area. A total of 16 health care facilities providing delivery service (1 general hospital, 2 primary hospitals, and 13 health centers) were selected for study using simple random sampling techniques from 32 health facilities in the eastern zone, health posts were not included in the study since they did not provide delivery service. All health care providers in the selected health care facilities who participated in immediate newborn care were involved in the study.

Observational questionnaire to assess the practice of immediate newborn care and interviewer guided structured and pre-tested questionnaire to assess knowledge of newborn care on Breastfeeding, immediate postnatal care, newborn infection management, care of low birth weight baby.

Ten-degree Midwives and Nurses were recruited for data collection and two BSc degree Nurse and four masters in midwifery as a supervisor. The overall data collection process was coordinated and supervised by supervisors, co-investigators and the principal investigator.

The validity of the questionnaire was maintained by adapting the questionnaire from different relevant kinds of literature that were used by other researchers and modified in the local context. 5% of the calculated sample size was pre-tested in another health facility which is not included in the study before the actual application for data collection. Data completeness and accuracy was checked on a daily basis by supervisors.

After the fieldwork completed and coded, then the data were entered and cleaned using SPSS version 20.0.

The data analysis methods used were descriptive analysis for frequencies, percentage, mean, standard deviation and percentage, Bivariate logistic regression analysis to see the relationship of each independent variable with practice of immediate newborn care and finally variables which are found significant at *p* < 0.05 in bivariate analysis was taken to multivariate logistic regression analysis to determine the final predictors with practice of immediate newborn care.

Model assumption fulfillment and multicollinearity test were done prior to multivariate logistic regression. The final logistic model goodness of fit was assessed using the Hosmer-Lemeshow goodness of fit test, an omnibus test of model coefficients and the result shows the model is fit.

Compute score was done to assess the result of knowledge and practice of health care providers involved in the study.

There were ten procedures to assess the immediate newborn care practice on observation (see Table 2) and operationally defined as.

*Good practice:* if the health care providers perform more than or equal to 70% the practice procedures.

*Poor practice*: if the health care providers performs less than 70% of the practice procedures.

There were 12 questions to assess knowledge of health care providers’ on immediate newborn care assessed using interview and operationally defined as.

*Adequate knowledge*: if the health care provider answers the knowledge question above or equal to mean.

*Inadequate knowledge*: – if the health care provider answers the knowledge questions below mean.

Finally, the result of the study was presented using tables and text.

## Result

### Socio-demographic characteristics

In this study 221 participants were eligible, but at the time of data collection six of the eligible study participants were not available due to office work and annual leave. Overall, among the eligible study participants, 215 were contacted, 2 interrupted between the interviews. A total of 213 participants has completed the survey, with a response rate of 99.1%, out of those 150 (70%) were females. The median age of the participants was 30.00 [IQR: 22–38] years. Approximately half of the participants were working in health center 116 (54.5%) and 44 (20.7%) of participants were trained in newborn care within the past two years. Moreover, the the majority of the participants 194 (91.1%) were Orthodox in religion and concerning the educational status of respondents 148 (69.5%) were diploma and 69 (29.1%) was degree. Working experience of the participants ranges from 1 to 39 years; median 5 years [IQR 3–12 years]. Other details are shown in Table 1 **.**

**Table 1:** Socio-demographic characteristics of healthcare providers in public health facilities at Eastern zone, Tigray regional state, Ethiopia, 2016

Variable		Frequency (N)	Percent (%)
Age (in year)	20–30	116	54.5
	31–40	71	33.3
	41–50	23	10.8
	≥51	3	1.4
Sex	Male	63	29.6
	Female	150	70.4
Religion	Orthodox	194	91.1
	Muslim	6	2.8
	Catholic	13	6.1
Educational status	Diploma	148	69.5
	Degree	62	29.1
	MSc	3	1.4
Monthly salary(USD)	75–108	79	37.1
	108.10–174	65	30.5
	174.10–207	41	19.2
	207.10–240	22	10.3
	≥240.10	6	2.8
Type health facility	General Hospital	56	26.3
	Primary Hospital	41	19.2
	Health Center	116	54.5
Working experience (in year)	1–5	111	52.1
	6–10	43	20.2
	11–15	17	8.0
	16–20	16	7.5
	21–30	25	11.7
	≥31	1	.5

### Practice of health care providers on immediate newborn care

The result of this survey showed that 155 (72.8%) of respondents generally have good newborn care practice; specifically 180 (84.5%) of the participants have properly practice putting a baby on to mother’s abdomen immediately after delivery, 184 (86.4%) of the participants place the baby in skin-to-skin contact and on the breast to initiate breastfeeding, 156 (73.2%) of participants apply Tetracycline eye ointment and 141 (66.2%) give Vitamin K IM on anterior mid-thigh but only 18 (8.5%) of participants apply chlorhexidine to cord after cord cutting **(**Table 2 **).**

**Table 2:** Practice of health care providers on Immediate Newborn Care in public health facilities at Eastern zone, Tigray regional state, Ethiopia, 2016

Variable		Frequency (N)	Percent (%)
Deliver baby on to mother’s abdomen	Yes	180	84.5
	No	33	15.5
Dry baby’s with dry towel, Wipe eyes, Wrap with dry one and cover head	Yes	184	86.4
	No	13	13.6
Assess breathing and color	Yes	187	87.8
	No	26	12.2
If the baby breath normally delay cord cutting 1–3 min	Yes	148	69.5
	No	65	30.5
Tie the cord two fingers from abdomen and another tie two fingers from the 1st one cut the cord between the 1st & 2nd tie	Yes	161	75.6
	No	52	24.4
Place the baby in skin-to-skin contact and on the breast to initiate B/feeding	Yes	184	86.4
	No	29	13.6
Apply chlorhexidine to cord after cord cutting	Yes	18	8.5
	No	195	91.5
Apply Tetracycline eye ointment once	Yes	156	73.2
	No	57	26.8
Give Vitamin K IM on anterior mid- thigh	Yes	141	66.2
	No	72	33.8
Weigh the baby	Yes	195	91.5
	No	18	8.5

### Knowledge of health care providers on immediate newborn care

Regarding knowledge of health care providers on option of breastfeeding of newborn 91.5% of the participants stated breastfeeding should start within one hour after delivery, 93% choice exclusive breastfeeding for six months option, 55.9% of participants stated breastfeeding should stop completely at two years from the duration of breastfeeding options and 78.4% of the participants choice breastfeed more frequently as option to the care of mother with inadequate breast milk during the first few days after delivery (See Table 3).

**Table 3:** Knowledge of health care providers about Breastfeeding of newborn baby in public health facilities at Eastern zone, Tigray regional state, Ethiopia, 2016

Variable		Freq. (N)	Percent (%)
Initiation of breastfeeding after birth	Within the first hour	195	91.5
	1–6 h after birth	12	5.6
	6–12 h	3	1.4
	More than 12 h after birth,	0.00	0.00
	Other	3	1.4
Mother inadequate breast milk during the first few days after delivery	Give formula while waiting for breast milk	19	8.9
	Breastfeed more frequently	167	78.4
	Give rice water, herbal fluids or honey water	8	3.8
	Advise the mother to ask someone else to breastfeed her baby	19	8.9
	Other	0.00	0.00
Period of exclusively breastfeed	2 months	4	1.9
	4 month	6	2.8
	6 months	200	93.9
	More than 6 months	2	0.9
	Other	1	0.5
After how long should a mother stop breastfeeding her child completely	24 months	119	55.9
	More than 2 years	78	36.6
	18 months	12	5.6
	6 months	3	1.4
	12 months	0.00	0.00
	I have no opinion	1	0.5

Regarding knowledge of health care providers in the immediate postnatal care of newborn babies, 38.97% of the participants have knowledge about prevention of newborn bleeding and 41.8% have knowledge about the correct dose of Vitamin K to be given to a term newborn and preterm baby. In addition, the Knowledge of health care providers about infection management of newborn baby shows 35.2% of the participants know how to apply eye drops (silver nitrate) after cleaning eyes to prevent eye infections after delivery and 87.3% of the participants have knowledge about the the care of umbilical cord after delivery. Other details are shown in Table 4 **.**

**Table 4:** Knowledge of health care providers about immediate postnatal care and Infection management of newborn baby in public health facilities at Eastern zone, Tigray regional state, Ethiopia, 2016

Variable		Freq. (N)	Percent (%)
Prevent newborn children from bleeding	Breastfeeding the child,	130	61.03
	Not necessary to give any drugs,	0.0	0.00
	Give Vitamin K	83	38.97
	I have no opinion,	0.0	0.00
Dose of Vitamin K to give to a term newborn baby	0.5 mg	102	47.90
	1 mg	89	41.80
	2 mg	4	1.90
	5 mg	0.0	0.00
	10 mg	1	0.50
	I have no opinion,	8	3.80
	Other	9	4.20
Prevention of eye infections after delivery	Do not apply anything	36	16.90
	Apply breast milk in the babies’ eyes	4	1.88
	Clean eyes with sterile gauze only	83	38.97
	Apply eye drops (silver nitrate) or TTC after cleaning eyes	75	35.20
	I have no opinion	4	1.88
	Other	10	4.7
Which care of the umbilical cord of newborn after delivery is important	Cut the cord with a clean instrument (for example, a razor, blade)	186	87.3 0
	Use any sharp instrument for cutting the cord	7	3.30
	After cutting the cord, apply for traditional herbs/medicines	1	0.50
	Always put a bandage on the cord,	8	3.80
	I have no opinion,	1	0.50
	Other	10	4.70

Health care providers’ knowledge about the care of a low birth weight baby, 75.6% of the study participants have knowledge on ways to stabilizing the temperature of a newborn baby, 81.2% of participants have knowledge of the definition of low birth weight of newborn child and 68.5% participants stated bath more frequently as not important when taking care of a low birth weight baby. Other details are shown in Table 5.

**Table 5:** Knowledge of health care providers about Care of a low birth weight baby in public health facilities at Eastern zone, Tigray regional state, Ethiopia, 2016

Variable		Freq. (N)	Percent (%)
Way to stabilizing the temperature of a newborn baby	Bathing the baby in water of appropriate temperature	6	2.80
	By putting on clothes and cover head,	0.0	0.00
	Having the baby skin-to-skin with her/his mother,	161	75.60
	Keep the baby in a room with a temperature of 28–30 °C,	15	7.00
	Have the baby close to heat (radiator, fire, etc.),	31	14.60
	Other	0.0	0.00
Definition of low birth weight for newborn child	Weight is less than 3000 g,	5	2.30
	Weight is less than 2500 g,	173	81.20
	Weight is less than 1500 g,	20	9.40
	Weight is less than 1000 g,	3	1.40
	I have no opinion,	7	3.30
	Other	5	2.30
Not important when taking care of a low birth weight baby	Start breastfeeding early and frequently	36	16.9
	Bath the baby often	146	68.5
	Keep the child warm	13	6.1
	Apply skin to skin contact	6	2.8
	Prevent infection from developing	4	1.9
	None	8	3.8

The overall knowledge of health care providers of Immediate Newborn Care in public health facilities of Eastern zone was 74.65%.

### Factors that affect the practice of immediate newborn care among health care providers

In bivariate logistic regression analysis age, sex, educational status, type of health facility, working experience, availability of national guideline, shortage of materials and the period of taking training were significant predictors but after controlling for the effects of potentially confounding variables using multivariate logistic regression only availability of national guideline, shortage of materials, the period of taking training and type of health facility were found to be significant predictors for practice of immediate newborn care. Those health facilities having adequate materials for newborn care were 4.69 times more likely practicing good newborn care compared to health facilities having a shortage of materials for newborn care [AOR; 4.69 95% CI: (1.5, 14.6**)]**. Similarly, facilities having a national guideline about newborn care were 28.4 times more likely practicing good newborn care than those did not have national guideline [AOR = 28.495% CI: (5.10,15.22)].

But those health care providers taking newborn care training before two years were 76% lower odds of newborn care practice compared to those taking training within two years [AOR = 0.2495% CI: (0.09,0.66)]. In addition, those health care providers working in health centers were lower odds of newborn care practice compared to health care providers working in General hospitals [AOR = 0.089, 5% CI: (0.01,0.65)]. For detail see (Table 6).

**Table 6:** Multivariate analysis to identify factors associated with Practice of health care providers on Immediate Newborn Care in public health facilities at Eastern zone, Tigray regional state, Ethiopia

Variable	Practice of newborn care		Cured OR (95% CI)	Adjusted OR (95% CI)
	No N (%)	Yes N (%)		
Work experience				
1–5	35 (60.3%)	76 (49.0%)	1.00	1.00
6–10	8 (13.8%)	35 (22.6%)	2.01 (0.84,4.79)	1.85 (0.28,11.95)
11–15	4 (6.9%)	13 (8.4%)	1.49 (0.45,4.92)	4.87 (0.24,97.20)
16–20	8 (13.8%)	8 (5.2%)	0.46 (0.16,1.32)	0.40 (0.02,6.63)
21–30	3 (5.2%)	22 (14.2%)	3.37 (1.23,12.03)*	9.6 (0.78,12.24)
≥ 31	0 (0.0%)	1 (0.6%)	2.17 (0.71,10.40)	24.4 (0.001,14.3)
Age				
20–30	24 (41.4%)	92 (59.4%)	1.00	1.00
31–40	28 (48.3%)	43 (27.7%)	0.40 (0.20.0.77)*	0.23(0.04,1.32)
41–50	6 (10.3%)	17 (11.0%)	0.73 (0.26,2.07)	0.21 (0.01,2.71)
≥ 51	0 (0.0%)	3 (1.9%)	3.80 (0.63,7.23)	7.29 (0.001, 15.1)
Shortage of material				
No	48 (82.8%)	46 (29.7%)	1.00	1.00
Yes	10 (17.2%)	109 (70.3%)	11.3 (5.3,24.4)*	4.69 (1.5,14.6)*
Guideline				
No	46 (79.3%)	15 (9.7%)	1.00	1.00
Yes	12 (20.7%)	139 (90.3%)	35.5 (15.5,81.3)*	28.4 (5.10,39.22)*
When do you take training				
Within two years	11(28.2%)	86 (79.6%)	1.00	1.00
Before two years	28(71.8%)	22 (20.4%)	0.10 (0.04,0.23)*	0.24 (0.09,0.66)*
Education				
Diploma	51(87.9%)	97 (62.6%)	1.00	1.00
Degree	7(12.1%)	55 (35.5%)	4.13 (1.75,9.72)*	1.74 (0.27, 10.89)
MSc	0 (0.0%)	3 (1.9%)	1.9 (0.67,5.24)	1.04 (0.04, 8.23)
Sex				
Male	10(17.2%)	53 (34.2%)	1.00	1.00
Female	48(82.8%	102(65.8%)	0.40 (0.18,0.85)*	0.39 (0.06,2.57)
Type of health				
General hospital	1 (1.7%)	63 (40.6%)	1.00	1.00
Primary hospital	2 (3.4%)	57 (36.8%)	0.45 (0.04,5.12)	0.32 (0.28.3.78)
Health center	55 (84.8%)	35(22.6%)	0.01 (0.001,0.76)*	0.08 (0.01,0.65)*

* = *P* ≤ 0.05 (* indicate level of significance), 1 = Ref.cat

## Discussion

The first few hours since birth is the most crucial period in the life of an infant for further growth and development, which is largely determined by the quality of care that the newborn receives [20].

In this study an effort has been made to assess knowledge, the practice of newborn care and associated factors among health care providers in public health facilities of eastern zone, Tigray, Ethiopia.

Overall, 74.65% of the health care providers participated in the study was having adequate knowledge on newborn care. This result is more or less similar to the study conducted in Addis Ababa 2013 which was 68% [21] but it is higher than the study conducted in Egypt (52.2%), Uganda (46.5%), and Khartoum (50.6%) [22]. This difference might be due to the difference in the period of study and a slight difference in the data collection tools we used.

Knowledge of breastfeeding initiation was 91.5%, this finding is in line with a study conducted in Uganda which was 86.4% [13]. The findings of this study reveal that the knowledge of Exclusive breastfeeding and age of a child to stop breastfeeding are 11.2% and 9.9% respectively. This result is lower than the study conducted in Uganda (2011) which was 64%, 19.1% respectively [13]. This difference might be due to the difference in access to short-term training provided to health care providers on breastfeeding.

Our study showed that 71.2% of the health care providers were having knowledge on the immediate postnatal care of a newborn; compare to study conducted in Uganda which was reported 72.5% knowledge of health care provides its more or less similar finding [13]. But it is lower than the study conducted in Sudan 98% [9], this difference might be due to the gap capacity building like in-service training on a newborn. Similarly, in this study the health care providers’ knowledge of vitamin K was 41.8% this was more or less similar to study conducted in Uganda which was 49.7% [13]. But it is lower than the study conducted in Haryana India 76% [23]. These variations could be largely due to the difference in the educational level of the participants and access to newborn care training provided to health care providers.

Knowledge about low birth weight management (75.6%) among health care providers reported from this study was more or less similar to study conducted in Uganda 71% [13].

In this study practice of newborn care among health care providers was 72.77%: this result is more or less similar to the study conducted in Vietnam (64%) [1], Egypt (69.2%) [20] But this finding was much higher than the study conducted in Haryana, India, Khartoum Sudan, Addis Ababa which was 55%, 41.1%, and 30% respectively [13, 21, 24]. Differently, this finding was much lower than the study conducted in Tanzania which was 88.2%. This difference might be due to a difference in materials, type of health facility and variation in the study period.

From the multivariate logistic regression analysis availability of national guideline was found a significant predictor of newborn care practice [AOR = 28.495% CI: (5.10, 15.22)]. This result is different from Vietnam, which shows the availability of national guideline was not significantly associated with newborn care practice [1].

In addition, those health care providers working in health centers were lower odds of newborn care practice compared to health care providers working in General hospitals. This finding is different from the study conducted in Uganda, which shows the type of health facility has no significant association with newborn care practice [21].

Those health facilities having adequate materials for newborn care were 4.69 times more likely practicing good newborn care compared to health facilities having a shortage of materials for newborn care. This finding is consistent with the study done in Addis Ababa [21].

The odds of newborn care practice were less by 76% for those health care providers taking training before two years compared to those taking training within two years.

## Conclusion

Majority health care providers in the public health facilities eastern zone of Tigray have adequate knowledge on initiation of breastfeeding, cord care, but they have a knowledge gap on prevention of eye infection and prevention and management of early bleeding. Generally, among the health care providers who participated in this study, 74.65% had adequate knowledge of newborn care.

Even though 72.77% of the participants have adequate newborn care practice, but specifically the study participants showed a gap in the practice of vitamin K administration and application of chlorhexidine to cord after delivery.

The health care providers practice of immediate newborn care were affected by the availability of the national guidelines, shortage of materials for newborn care, the time of taking training on newborn care and type of health facility.

Based on findings of the study, we recommend to Tigray regional health bureau and district health offices in collaboration with other stakeholders: to strengthen providing refreshment in-service training on immediate newborn care regularly and follow up, equipping all health facilities with national guideline of newborn care and necessary materials to provide immediate newborn care and there should be sharing experience between Hospital staffs and health centre staffs working on newborn care through mentoring.

## Abbreviations

AOR: Adjusted odd ratio
EDHS: Ethiopia demographic and health surveys
ENC: Essential newborn care
IM: Intramuscular injection
IQR: Inter quartile range
MDG: Millennium development goal
SPSS: Statistical package for social science
TTC: Tetracycline
WHO: World Health Organization
